# Clinical, morphological and genetic features of a cohort of late onset GSD II patients: typical and atypical presentations

**DOI:** 10.1186/1471-2474-14-S2-P9

**Published:** 2013-05-29

**Authors:** E Barca, O Musumeci, C Rodolico, A Ciranni, G Vita, A Toscano

**Affiliations:** 1 Department of Neurosciences, University of Messina, Messina, Italy

## Introduction

GSDII (Pompe disease) is a rare, autosomal recessive disease due to alpha-glucosidase (GAA) deficiency that presents with infantile and late-onset forms. Herein, we describe a cohort of 29 late-onset patients (15 males and 14 females, aged 9 to 76), and discuss some unusual clinical features seen in 4 of them.

## Methods

Clinical evaluations were performed in all patients using the Walton and Gardner-Medwin, MRC and GSGC scales, and the 6MWT. Respiratory function was assessed using FVC% in upright and supine positions. All patients underwent morphological and biochemical examinations, muscle MRI, neurophysiological studies, and molecular genetic analysis.

## Results

Out of 29 patients, 15 received enzyme replacement therapy (ERT). 57% initially had limb-girdle involvement, 29% an isolated hyperCKemia, and 14% respiratory insufficiency. Neurophysiological studies revealed a myopathic pattern in 48% of patients, neurogenic or mixed in 28%, whereas 24% of patients did not show any electrical abnormality. In the examined patients, muscle MRI showed early involvement of ileopsoas, gluteus and posterior thigh muscles. Morphological studies revealed vacuolar myopathy in 68% of patients, and biochemical analysis showed residual alpha-glucosidase activity ranging from 0.01% to 25%. Molecular genetic analysis confirmed IVS13T>G as the most common mutation, but we also identified three novel point mutations. Atypical clinical features were observed in 4 out of 29 patients: a 66-year-old female with disease onset involving distal upper limbs muscles; a 70-year-old male with mesial temporal sclerosis without epileptic or cognitive disorders; a 46-year-old male with severe neurosensory hearing loss (with very early hearing difficulties); and a 51-year-old female with a congenital absence of a thyroid lobe.

## Conclusion

Our study confirms the clinical, morphological and molecular genetic heterogeneity of late-onset GSDII. Muscle MRI is a very useful tool for muscle damage evaluation, especially at the early stages of the disease. Based on our experience with some atypical cases, we suggest always performing a complete clinical and laboratory evaluation of patients in order to highlight unusual muscular (e.g. distal involvement) and/or CNS presentations. These cases reinforce the hypothesis that modifier genes and/or epigenetic factors may contribute to clinical presentations of Pompe disease.

